# Acute kidney injury is associated with elevated urinary endotrophin

**DOI:** 10.1152/ajprenal.00300.2025

**Published:** 2025-10-13

**Authors:** Amanda J. Clark, Brenda Mendoza Flores, Marie Christelle Saade, Kyle Q. Vu, Isaac J. Pence, Ningyan Zhang, Zhiqiang An, Dawei Bu, Philipp E. Scherer, Samir M. Parikh

**Affiliations:** 1 Division of Pediatric Nephrology, Department of Pediatrics, University of Texas Southwestern, Children’s Medical Center Dallas, Dallas, Texas, United States;; 2 Division of Nephrology, Department of Internal Medicine, University of Texas Southwestern, Dallas, Texas, United States;; 3 Department of Biomedical Engineering, University of Texas Southwestern, Dallas, Texas, United States;; 4 Texas Therapeutics Institute, Brown Foundation Institute of Molecular Medicine, University of Texas Health Science Center at Houston, Houston, Texas, United States;; 5 Touchstone Diabetes Center, University of Texas Southwestern, Dallas, Texas, United States;; 6 Department of Pharmacology, University of Texas Southwestern, Dallas, Texas, United States

**Keywords:** acute kidney injury, biomarker, endotrophin, metabolism

## Abstract

Acute kidney injury (AKI) is prevalent among hospitalized patients. Novel biomarkers are needed to diagnose AKI and target therapies. Endotrophin (ETP) is a molecule released during collagen type VI formation that may promote injury and fibrosis. Although serum ETP elevation has been associated with adverse outcomes in AKI, urinary ETP has not been assessed in AKI, nor has ETP been evaluated in a pediatric population. Urine samples were collected from a tertiary children’s hospital. Medical records were reviewed, and patients who met criteria were sorted into three categories: *1*) AKI; *2*) hospitalized controls; and *3*) outpatient controls. ETP was measured using ELISA, and results were corrected to urine creatinine (uETP:Cre). A multivariate linear regression assessed whether demographic variables were independently associated with uETP:Cre. Odds of AKI were assessed in serial uETP:Cre tertiles using a multivariate logistic regression model that adjusted for patient variables. uETP:Cre was elevated in patients with AKI compared with hospitalized patients without AKI (*P* < 0.05) and outpatient controls (*P* < 0.0001). Multivariate analysis revealed that age, but not sex, race, or ethnicity independently correlated with uETP:Cre. After adjustment for these variables, the odds ratio for AKI increased with serial uETP:Cre tertiles. Noninvasive measurement of uETP may deliver meaningful information to aid AKI diagnosis. Given that ETP may be both a biomarker and a clinically actionable stimulus of inflammation and fibrosis, future studies are needed to understand the role of elevated ETP in AKI and whether existing ETP-neutralizing antibodies could represent a new avenue of AKI therapy.

## NEW & NOTEWORTHY

Endotrophin (ETP) is a molecule released during the formation of type VI collagen that may promote fibrosis and inflammation. Serum ETP is elevated in acute kidney injury (AKI) and associates with adverse outcomes. Urine ETP during AKI has never been assessed. For the first time, this study demonstrates that urine ETP is also elevated during episodes of AKI, representing a novel, noninvasive AKI biomarker that may be clinically actionable.

## INTRODUCTION

Acute kidney injury (AKI) is estimated to affect 21% of hospitalized adults (1) and 11%–31% of hospitalized children (2–4) in the United States with significant associated costs (5, 6) and mortality (1). Current diagnostic criteria for AKI depend on incremental changes in serum creatinine and urine output, which lead to inaccurate and delayed AKI diagnosis. There is no treatment for AKI beyond supportive measures. Novel biomarkers are needed to diagnose AKI more rapidly and accurately and also target AKI therapies.

Endotrophin (ETP) is a signaling molecule released during the formation of collagen type VI. In certain clinical settings, it acts as a pathogenic growth factor and fibrogenic stimulus (7–9). Endotrophin has also emerged as candidate biomarker in numerous inflammatory conditions including breast cancer, pancreatic carcinoma, colorectal cancer, hepatocellular carcinoma, diabetes, lung disease, heart failure, and liver disease, where it distinguishes affected patients from controls and also associates with disease severity and mortality (10). In the kidney, serum and urine ETP (uETP) have been associated with fibrosis severity and kidney function in IgA nephropathy and antineutrophil cytoplasmic antibodies (ANCA) vasculitis (11). In a diverse CKD population, uETP elevation was associated with CKD progression and mortality (12, 13). Among patients with AKI, serum ETP was elevated compared with patients without AKI, and ETP correlated with major adverse kidney events (MAKE) (14). Even 1 yr after AKI, affected individuals exhibited higher plasma ETP compared with patients who had not experienced AKI, and ETP levels were associated with disease progression (15).

ETP may not only act as a biomarker of disease, but a therapeutic target. Neutralizing antibodies against ETP have been shown to decrease tumor growth in mice (16), liver fibrosis in cell models (17), and renal fibrosis in a murine model of focal segmental glomerular sclerosis (FSGS) (18). Neutralizing antibodies have not yet been tested in AKI models. Likewise, urinary ETP has not been assessed in AKI nor has ETP ever been evaluated in a pediatric population. Learning whether noninvasive urine measurements of ETP may be meaningful in AKI diagnosis and pathogenesis, and whether ETP may also be pathogenic in pediatric disease, will be critical to expand the understanding of ETP biology and maximize therapeutic potential of ETP-directed therapies.

## MATERIALS AND METHODS

### Ethics

This study was approved by the institutional review board at University of Texas Southwestern (STU-2021-0835) and was carried out in accordance with the Declaration of Helsinki. This study was performed on discarded samples with minimal risk to patients; thus, no informed consent was obtained.

### Study Population and Sample Collection

Samples presented here are a reanalysis of a previously published cohort (19). In brief, all urine samples discarded from a tertiary care children’s hospital were collected during a 4-mo period. Electronic medical records were reviewed, and patients who met criteria were sorted into three categories: *1*) hospitalized patients in ICU or on the inpatient floor with AKI at the time of collection; *2*) hospitalized control patients who were admitted to the ICU or inpatient floor with no AKI; and *3*) outpatient controls who had urine samples sent for routine screening tests, such as STI or pregnancy testing. Within the AKI group, patients were subclassified into prerenal or intrinsic AKI via independent review of the medical record by a pediatric nephrologist.

### Data Collection

Patient age, race, and sex were collected from the electronic medical record. eGFR was calculated using the bedside Schwartz equation. Urine creatinine was measured using a commercial colorimetric assay (Bioassay Systems DICT-500). Endotrophin was measured using an ELISA, as previously described (14). In brief, microwell plates were coated with rabbit monoclonal anti-ETP antibodies. Urine was added at 1:10 and 1:20 dilutions in PBS. High-affinity anti-ETP (ETN-1Rb) was used as a secondary antibody, and anti-rabbit Fab2-horseradish peroxidase antibody (Jackson ImmunoResearch) was used for detection. Purified ETP recombinant protein was used in serial dilutions to create a standard curve.

### Data Processing and Statistical Analysis

Urine creatinine was used to normalize ETP to urine concentration. For statistical comparison, continuous variables were compared with Mann–Whitney test, and correlations were assessed with a coefficient of determination. Grubb’s test was used to detect and remove outliers with a *Q* = 0.1%, so that only definitive outliers would be removed. A multivariate linear regression was implemented in R (version 4.5.1) to assess whether demographic variables independently associated with uETP:Cre. Odds of AKI were assessed in serial uETP:Cre tertiles using multivariate logistic regression models that adjusted for patient variables.

## RESULTS

**Figure 1.**
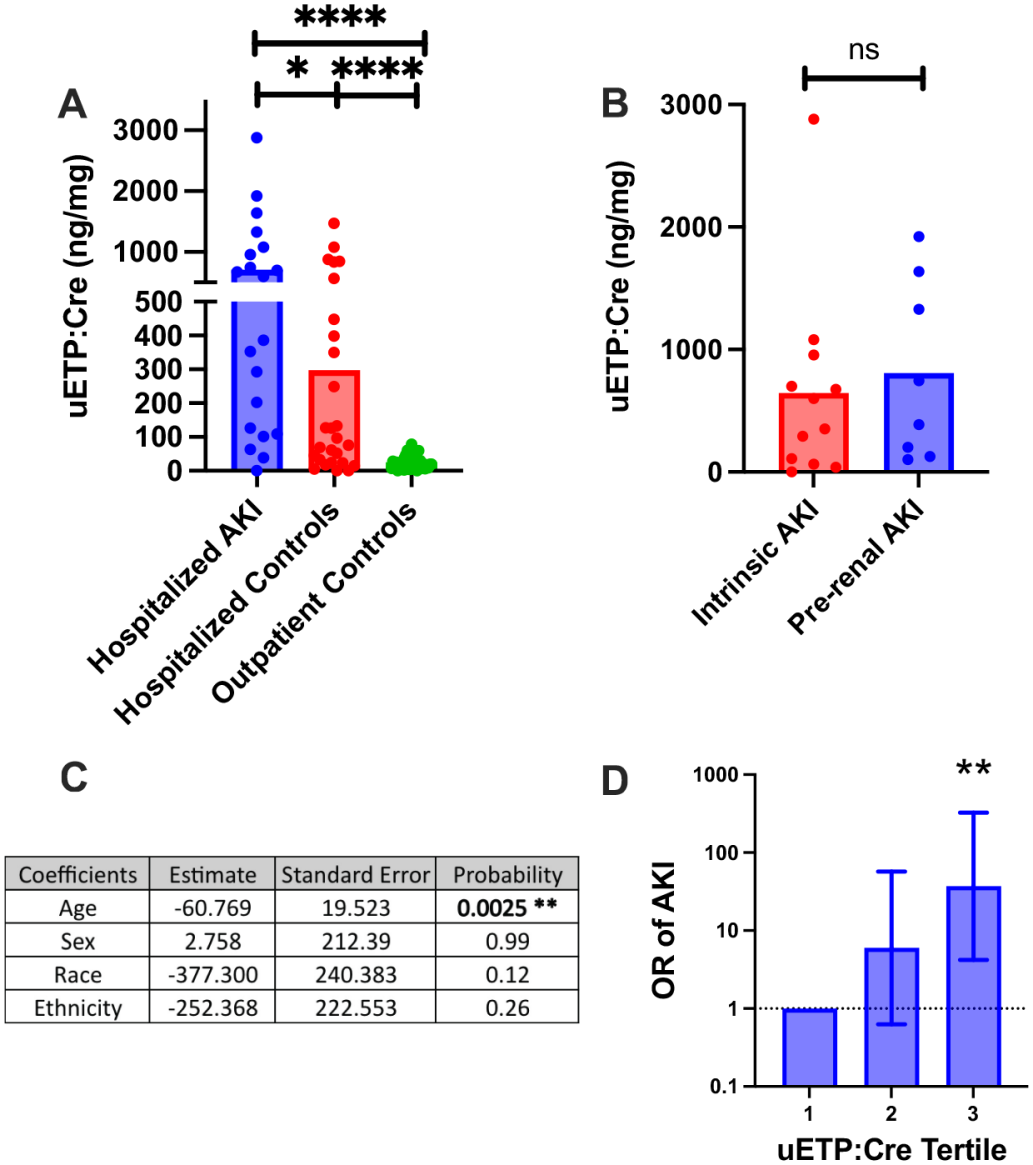
uETP:Cre in patient groups (*A*), uETP:Cre in patients with AKI separated by AKI etiology (*B*), Results of multivariate linear regression (*C*), OR of AKI in serial tertiles of uETP:Cre (*D*). AKI, acute kidney injury; Cre, urine creatinine; OR, odds ratio; uETP, urine endotrophin, *P < 0.05, **P < 0.01, and ****P < 0.0001.

A total of 39 outpatient control patients, 30 hospitalized control patients, and 21 patients with AKI were evaluated. There was no significant difference in age, sex, or race between groups (Table 1). Outpatient control patients had an average urinary endotrophin:creatinine ratio (uETP:Cre) of 25.23 ng/mg ± 2.257. Hospitalized control patients had a mean uETP:Cre of 297.5 ng/mg ± 76.25. AKI patients had a mean uETP:Cre 710.0 ng/mg ± 168.2 (Fig. 1A). The hospitalized control group had higher uETP:Cre compared with outpatient controls (*P* < 0.0001). The AKI group had higher uETP:Cre than both the control group (*P* < 0.0001) and the non-AKI hospitalized group (*P* = 0.02, Fig. 1A).

Among all patients with AKI, there was no correlation between uETP:Cre and eGFR (*R*^2^ = 0.04, not shown). There was no difference in uETP:Cre between patients with intrinsic or prerenal AKI (*P* = 0.43, Fig. 1B).

A multivariate linear regression determined that patient age independently associated with uETP:Cre when controlling for all other demographic variables, with younger age associated with higher uETP:Cre (*P* = 0.0025, Fig. 1C). Multivariate logistic regression showed that odds of AKI diagnosis increased across serial tertiles of uETP:Cre when controlling for all demographic variables (Fig. 1D).

## DISCUSSION

This cross-sectional evaluation of uETP among children with and without AKI demonstrates for the first time to our knowledge that urine ETP is elevated in AKI compared with controls. Our results suggest that noninvasive urine measurement of ETP may deliver clinically meaningful information that could aid in AKI diagnosis.

In adult kidney disease, serum ETP is elevated in AKI and associated with poorer outcomes (14). Persistent elevation of plasma ETA corresponded with poor recovery of renal function after AKI (15). The results presented here demonstrate that uETP may perform similarly to blood measurements of ETP, offering a less invasive monitoring option. Additional prospective studies will need to determine how closely urine and blood measurements correlate in the AKI setting and if urine measurements associate with clinical outcomes and disease severity as serum and plasma measurements have been shown to do.

Also of interest is the potential of uETP to act as an orthogonal biomarker providing distinct but complementary information to existing markers. This is most pronounced in the elevation of uETP among patients with both prerenal and intrinsic AKI, a notable distinction from prominently used clinical biomarkers like urinary neutrophil gelatinase-associated lipocalin (NGAL), which is elevated uniquely during intrinsic injury (20). This may relate to the physiologic mechanism behind increased ETP production in periods of stress. Collagen VI is produced and excreted in an inactive form primarily by fibroblasts. In response to unknown proinflammatory signals, multiple enzymes cleave extracellular collagen VI into its active cleavage products, which include ETP (21). This is in contrast to NGAL, which is expressed and excreted by renal tubule cells in response to injury (20). The rapid production of ETP from posttranslational cleavage may indicate its potential as a very early marker of kidney stress. Our data also demonstrate a poor correlation between uETP and eGFR and the elevation of uETP in patients who are hospitalized without AKI compared with controls. Although previous studies have shown a correlation between uETP and eGFR in patients without AKI (22), the heterogeneous nature of AKI in this population and the difficulty in estimating GFR in pediatric patients using serum creatinine make this finding unsurprising. Additional prospective studies are needed to determine when uETP rises in the course of AKI and whether it offers supplementary clinical information above existing biomarkers.

Finally, in animal models, ETP is not only a biomarker but also a stimulus of inflammation and fibrosis formation (7–9). This is meaningful as a therapeutic neutralizing antibody exists that has shown promise in animal models (16–18). Future studies will be needed to understand the role of elevated ETP in AKI and whether targeted ETP-neutralizing antibodies could represent an exciting new avenue of AKI therapy.

A limitation of this study is the sampling method, which utilized discarded patient samples. This led to a heterogeneous sample population with multiple stages of AKI represented at various stages of worsening or improving injury. However, the ability to detect a robust uETP elevation in patients compared with controls despite this heterogeneity highlights the prominence of uETP as a marker of AKI. Unfortunately, the small sample size does not allow subgroup analysis to assess uETP elevation in different stages of AKI or in relation to clinical outcomes. Future prospective studies will be needed in this regard. Interestingly, our data demonstrated an independent association between uETP and age, with younger age associated with higher uETP. This has not been previously reported, nor has ETP been measured in a pediatric population to our knowledge. Although age-correction still demonstrated elevated uETP in AKI, the underlying effect of age on ETP warrants additional study, perhaps examining whether periods of growth, puberty, or rapid cell turnover may increase ETP. Another unanswered question is how to distinguish uETP elevation from AKI versus other stressors. This can be demonstrated in our hospitalized control group, where patients had elevated uETP:Cre compared with outpatient controls but decreased uETP:Cre compared with patients with AKI. It is possible that hospitalized patients “without AKI” had underlying AKI from their condition that was not detected by creatinine alone. It is also possible that ETP elevation, a product of fibroblast activation, is a marker of systemic inflammation, and uETP may serve as more of an AKI risk assessment marker rather than a direct marker of intrinsic kidney injury. At 10–15 kDa in size, endotrophin should freely filter through the glomerular filtration barrier. Additional studies will be needed to decipher whether uETP from AKI can be distinguished from other sources of uETP elevation, whether uETP offers additive clinical information from plasma ETP and other known clinical and chemical biomarkers, and whether prospective measurement of uETP could predict kidney injury or other clinical outcomes.

## Figures and Tables

**Table 1.** Patient demographic variables

	Number of Patients	Mean Age, yr	Female n (%)	White n (%)	Mean eGFR (mL/Min/1.72 m^2^)
Outpatient controls	39	10.7	18 (45)	24 (60)	96
Hospitalized controls	30	9.3	16 (53)	18 (60)	121
Acute kidney injury	21	10.2	10 (48)	10 (48)	66*

* Excludes patients receiving renal replacement therapies.

## Data Availability

Data will be made available upon reasonable request.
